# Male Armaments and Reproductive Behavior in “Nutcracker” Camel Crickets (Rhaphidophoridae, *Pristoceuthophilus*)

**DOI:** 10.3390/insects6010085

**Published:** 2015-01-07

**Authors:** Lauren P. Conroy, David A. Gray

**Affiliations:** Department of Biology, California State University, Northridge, 18111 Nordhoff Street, Northridge, CA 91330-8303, USA; E-Mail: dave.gray@csun.edu

**Keywords:** male-male competition, forced copulation, weaponry, comparative study, Orthoptera

## Abstract

Males of many species possess striking weaponry used in intrasexual competition for access to females. Until recently, there were no known cases of male weaponry being used against females in sexual coercion. However, in the camel cricket, *Pristoceuthophilus marmoratus*, males use modified hind legs to fight with each other and also to trap females and force them to copulate. To determine whether hind leg armaments serve similar fighting and mating functions in morphologically similar congeners, we performed a comparative survey of armament use in intra- and inter-sexual interactions in four additional species of *Pristoceuthophilus* (*P. arizonae* and three undescribed species: *P.* ‘Huachuca summer,’ *P.* ‘Madera’ and *P*. ‘Mt. Pinos’). Intrasexual leg fighting occurred in all species for which trials were performed, and hints of sexual coercion occurred in two species (*P.* ‘Huachuca summer’ and *P.* ‘Mt. Pinos’), suggesting additional cases of a uniquely dual-purpose armament. These findings suggest an evolutionary exaptation of hind leg armaments in this genus, wherein an intrasexual fighting weapon took on a secondary function of sexual coercion.

## 1. Introduction

Agonistic interactions are well documented among conspecific males vying for access to females. Often, these fights are facilitated by male-specific weapons that are absent in females. Examples of these weapons are found in a wide range of animal taxa, with horns, antlers, spines and tusks arming the ranks of rhinoceroses and rhinoceros beetles alike [1]. The fighting advantage conferred by these weapons maintains their expression in a population; thus, armaments are primarily a result of intrasexual selection [2], although they often also serve as a basis for female assessment of male quality [3].

**Figure 1:**
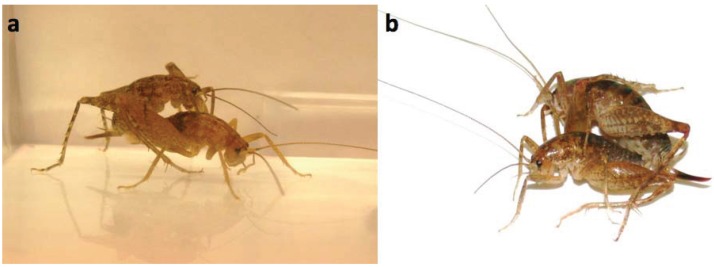
Comparison of voluntary (**a**) and forced copulation (**b**) in *P. marmoratus*. In voluntary copulation, the female is mounted on top of the male. In forced copulation, the male grabs the female with one or both hind legs and forcibly inserts his genitalia into hers while holding her on the ground, skewed to the side.

Although armaments play a major role in male-male combat, there is no evidence in the literature of their use in male-female interactions. Harassment of females and mating coercion occur in many species, but they are typically mediated by brute force associated with a larger male body size [4,5] or by specialized coercive structures, such as the abdominal “gin trap” of sagebrush crickets, *Cyphoderris strepitans* [6], modified anal cerci in bush crickets [7,8] and grasping antennal hooks in water striders, *Rheumatobates rileyi* [9], that lack a role in male-male interactions. Haley and Gray’s [10] study of *Pristoceuthophilus marmoratus* Rehn, 1904, was the first to reveal a male armament, modified male hind legs, that functions in both intrasexual and intersexual interactions. In adult males, the hind legs are modified with bent tibia and enlarged femoral spines; these leg modifications facilitate grasping and leg wrestling male opponents and occasionally grasping and capturing females for mating (Figure 1; see the videos at http://onlinelibrary.wiley.com/doi/10.1111/j.1439-0310.2011.01985.x/suppinfo). (N.B., in many species of leaf-footed bugs (Hemiptera: Coreidae), males use their spiny hind legs in a functionally similar manner to grapple with other males, but not to capture females [11,12].)

It appears to us that male leg use with females in a mating context is coercive; however, we cannot completely rule out the possibility that females gain information about male size and/or quality via assessment of potential mates during mating struggles. We interpret it as coercion rather than assessment because: (1) females often do not resist males once “embraced” by the male hind legs, which is more consistent with convenience polyandry to minimize the costs of resistance than with assessment (assessment requires active female resistance, otherwise even the weakest males would pass the test) [13]; (2) the use of legs in a mating context in *P. marmoratus* appears likely to be a condition-dependent alternate male strategy; smaller males are more likely to attempt to capture and control females for mating than larger males [14]; this is despite the fact that the leg modifications are positively allometric [10], such that large males would be the best equipped to capture females and presumably most likely to overcome female resistance during an assessment test.

Given the novelty of this “dual-purpose” structure, we undertook a comparative and primarily descriptive study to further explore how widespread these hind leg modifications might be among congeners and to attempt to observe how males use their legs in both intra- and inter-sexual interactions. The purpose of this study was thus to examine morphology and behavior in several congeneric species: (1) to describe statistically the morphological features of male armature and their allometric scaling with body size; (2) to observe male-male fighting behavior and male-female mating behavior; and (3) to make an initial attempt to infer the evolutionary origin of coercive mating behavior using modified hind legs in the genus. Allometric scaling can be informative about the form and strength of selection on a trait [15,16,17]; as mentioned above, the male hind leg modifications are positively allometric in *P. marmoratus* [10], suggesting strong selection for added investment. Inferring the evolutionary origins of traits is always complicated and is best conducted in a phylogenetic framework with a known phylogeny and dense taxon sampling. This is not possible at this time in this study system, because there is no published comprehensive taxonomic treatment of the group, let alone a well-resolved phylogeny. Nonetheless, we think some inferences can be made, and for taxonomic expertise, we relied heavily on correspondence with the late Dr. T. J. Cohn, who had worked on the group for many decades, but unfortunately died in 2012 without publishing his extensive materials. Because of this situation, we describe below in some detail the taxa used, which include three undescribed species, one of which was unknown even to Dr. Cohn.

## 2. Experimental Section

### 2.1. Taxonomic Issues and Species Status

*Pristoceuthophilus marmoratus* is one of about twenty species that share the male modified hind leg armaments described by Haley and Gray (enlarged femur, femoral spines, bent tibia). These species were grouped by Cohn into an unofficial subgenus, “Nucifractor,” or nutcracker, in reference to the way that males sometimes leg-squeeze a person’s finger while being handled [18]; adult males of many other species of *Pristoceuthophilus* do not have the hind leg modifications described here and are not considered “nutcrackers”, although the monophyly of “Nucifractor” has not been formally established. Most of these “nutcracker” species are undescribed, although the late T. J. Cohn and the even later T. H. Hubbell amassed a great store of morphological and geographic range descriptions about them.

**Figure 2:**
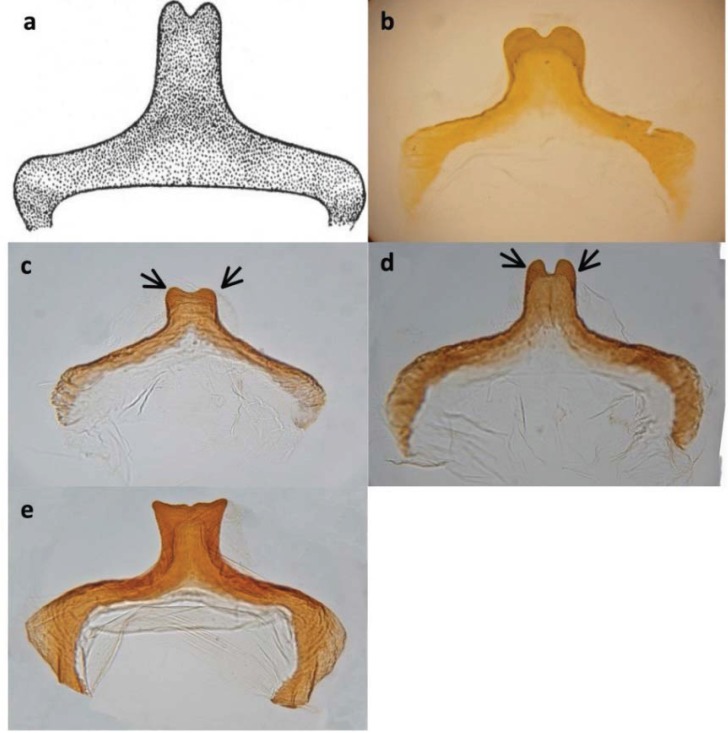
Caudal view of adult male pseudosternites from (**a**) the published *P. arizonae* description [19], (**b**) the *P. arizonae* reference specimen from the University of Michigan Museum of Zoology, (**c**) the *P. arizonae* specimen collected from Reef Townsite in October, 2012, (**d**) the putative new species, *P*. ‘Huachuca summer,’ collected from Reef Townsite in July, 2013, and (**e**) *P.* ‘Madera’ from the Santa Rita Mountains, AZ. Although similar, the pseudosternites of *P. arizonae* and *P.* ‘Huachuca summer’ are consistently distinguishable in that *P. arizonae*’s two bumps (see arrows) are more symmetrically rounded than those of* P*. ‘Huachuca summer’, and the groove between them is shallower in *P. arizonae.* (**b**–**e**) by J. N. Hogue.

**Figure 3:**
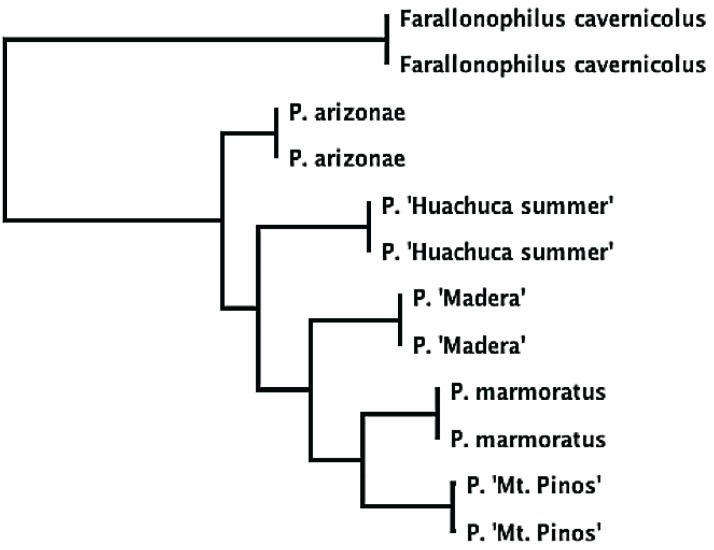
Phylogeny of the *Pristoceuthophilus* species used in this study, based on maximum likelihood analysis of 560 base pairs of the 16s ribosomal RNA gene (mitochondrial). Two samples were taken from each species. *Farallonophilus cavernicolus* Rentz 1972, another camel cricket in tribe Pristoceuthophilini, is the outgroup [20]).

Of the about twenty nutcracker species, we initially selected three on the basis of accessibility and our ability to identify them. Collection sites were recommended by T. J. Cohn[18]. In 2013, we collected an undescribed species (“*P. dasyglossus*” is the manuscript name in Hubbell and Cohn’s notes) from McGill Campground on Mt. Pinos, Los Padres National Forest, Kern County, California; we refer to it here as *P.* ‘Mt. Pinos.’ The other two species we collected in Arizona, where Cohn had told us that only two species of *Pristoceuthophilus* existed: an undescribed mid-elevation species (“*P. leptodrilus*” is the manuscript name in Hubbell and Cohn’s notes) and a high elevation species, *P. arizonae* Hebard 1935. In 2013, we collected the mid-elevation species “*P. leptodrilus*” from lower Madera Canyon in the Santa Rita Mountains, Coronado National Forest; here, we refer to this species as *P*. ‘Madera.’ A preliminary collecting trip in late September, 2012, to the high elevation Reef Townsite Campground in the Huachuca Mountains, Coronado National Forest, yielded a handful of adult male *P. arizonae*. However, on a return trip to the same site in July, 2013, we discovered adult male *Pristoceuthophilus* crickets that were a distinctly different color from the 2012 *P. arizonae* specimens; the 2012 *P. arizonae* specimens were a dull gray, whereas the 2013 July specimens were a deep mahogany-brown. As Dr. Cohn had informed us that only two species of *Pristoceuthophilus* occurred in Arizona, we considered the possibility that we had discovered a species unknown to either him or Hubbell, which we refer to here as *P.* ‘Huachuca summer.’ Unfortunately, Dr. Cohn died in 2012, so we could not consult his opinion on the matter; however, additional morphological (Figure 2; also see the Results), as well as genetic comparisons (Figure 3) support the distinctness of the two phenologically isolated morphs from Reef Townsite Campground. (All specimens gathered by us were stored in the collection of D.A. Gray at California State University, Northridge. Specimen information is available upon request.)

### 2.2. Collection and Housing 

The collection of all five species was accomplished by nocturnal checks of oatmeal piles. Adults were collected for all species, but *P.* ‘Mt. Pinos,’ for which juveniles were collected. From July 25–26, 2013, *P.* ‘Huachuca summer’ adults (*n* = 80 males, 103 females) were collected from mixed conifer forest in Reef Townsite Campground in the Huachuca Mountains in Coronado National Forest (Cochise County, Arizona; 31.43°N, 110.29°W; elevation, 2,180 m). (Undifferentiated juvenile *P. arizonae* (*n* = 3) were also collected at the same time and at the same oatmeal piles as *P.* ‘Huachuca summer’ adults; one *P. arizonae* individual survived to adulthood in the lab in November, 2013.) On September 29, 2012, *P. arizonae* adults (*n* = 12 males, 4 females) were collected from the exact same trail in Reef Townsite Campground. *Pristoceuthophilus* “Madera” adults (*n* = 32 males, 24 females) were collected from October 3–5, 2013, from riparian woodland in Madera Canyon in the Santa Rita Mountains in Coronado National Forest (Pima County, Arizona; 31.71°N, 110.87°W; elevation, 1,500 m). From October 1–2, 2013, *P. marmoratus* adults (*n* = 113 males, 69 females) were collected from riparian woodland in Malibu Creek State Park in the Santa Monica Mountains (Los Angeles County, California; 34.10°N, 118.73°W; elevation, 200 m). *Pristoceuthophilus* “Mt. Pinos” early, sex-differentiated juveniles (*n* = 108 males, 98 females) were collected from July 1–2, 2013, from mixed conifer forest in McGill Campground on Mt. Pinos in Los Padres National Forest (Kern County, California; 34.81°N, 119.10°W; elevation, 2,300 m).

Upon collection, crickets were individually housed in a 27 °C growth room on a 12:12 h dark:light reversed photoperiod. Each individual was placed in a 500-mL plastic tub with a cotton-plugged water vial, *ad libitum* Purina Cat Chow and egg carton pieces for shelter. *Pristoceuthophilus* “Mt. Pinos” juveniles were reared collectively in communal tubs (50–100 crickets per tub) until adulthood, at which point they were isolated into individual tubs.

Note: sample sizes were somewhat uneven across species, with especially small collections of *P. arizonae* and *P.* ‘Madera’ individuals due to imperfect information about the adult maturation periods of these species.

### 2.3. Measurement of Body Size, Armaments and Allometry

**Figure 4:**
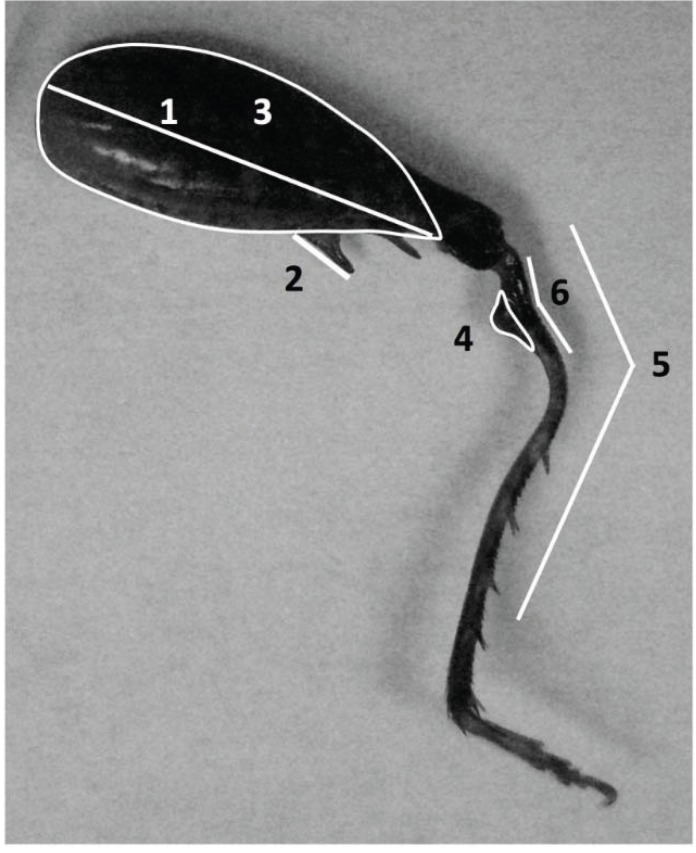
Hind leg of a *P.* ‘Huachuca summer’ adult male showing the traits measured. 1 = femur length, 2 = spine length, 3 = femur area, 4 = flange area, 5 = primary angle of tibial deflection, 6 = secondary angle of tibial deflection. *P. marmoratus* lacks the flange (4) and the secondary tibial deflection (6).

Male mass was measured immediately before behavioral trials (see below). Measurements of pronota and hind leg traits were made after crickets were preserved in ethanol (*P.* ‘Huachuca summer’ *n* = 51, *P. arizonae* *n* = 12, *P.* ‘Madera’ *n* = 13, *P. marmoratus* *n* = 101, *P.* Mt. Pinos *n* = 61). The pronotum length along the midline to the femoral joint was measured with digital calipers. Hind legs were photographed medial side down, and ImageJ was used to measure five traits: maximum length of proximal femoral spine, femur area, primary and secondary angles of tibial deflection and flange area (Figure 4). (N.B., *P. marmoratus* lacks a secondary tibial deflection and a flange; hence, these traits were not measured for this species.) Femur area was measured by excluding the femoral joint and tapering to the end of the pronotum length measurement. The primary angle of tibial deflection was determined by measuring the angle from the proximal tibia to the third distal tibial spur and subtracting that angle from 180°. The secondary angle of tibial deflection was determined by measuring the angle from the proximal tibia to the primary tibial bend and subtracting that angle from 180°. The flange area was determined by including the total area that bulged from the ventral line of the tibia. Each pronotum and hind leg trait was measured twice, and the average of the two values was used in analyses. Measures of allometric slopes were made by regressing the six traits on pronotum length using reduced major axis (RMA) regression (after square root-transforming areas and arcsine square root-transforming angles).

### 2.4. Behavioral Trials: Male-Male Fights and Male-Female Mating

Behavioral trials took place at least two weeks after field collection to ensure full sexual maturity and elimination of individuals harboring tachinid larvae [21]. In each species, except for *P. marmoratus*, male-male fighting trials and male-female mating trials were performed (in *P. marmoratus*, male-male fighting data were taken from [10]). Male-male trials consisted of two males plus one female for incentive, while male-female trials consisted of one male and one female. Individuals were randomly grouped or paired for trials. Each individual was used only once in a male-male trial or a male-female trial, but some individuals were used in both a male-male trial and a male-female trial (although they were never matched with the same individual(s) from the previous trial). When an individual was used in both trial types (*P*. ‘Huachuca summer’ *n* = 0 males, 0 females; *P*. ‘Madera’ *n* = 11 males, 3 females; *P. marmoratus* = *NA*; *P.* ‘Mt. Pinos’ *n* = 55 males, 18 females), the male-male trial always preceded the male-female trial, with a minimum 2-day latency between trials.

Behavioral trials were conducted at 22 °C during the dark portion of the crickets’ photoperiod. Each trial was preceded by a thirty-minute acclimation period to allow the crickets to adjust to the colder temperature (which more accurately simulated autumn nighttime conditions). Three-hour observation trials took place in a 14 × 8 × 10 cm (*l* × *w* × *h*) plastic tub under a red light, to minimize light disturbance; up to 6 trials in 6 tubs were conducted simultaneously. We noted the crickets’ behavior while minimizing movement and noise and recorded whether or not a fight took place (male-male trials only) and the presence or absence of any mating, voluntary or forced (all trials). A fight was recorded if males grappled with their hind legs and attempted to pin their opponent in a leg lock. Voluntary* versus* forced mating were easily distinguishable: in voluntary copulation, the female climbs onto the male’s back and is not in contact with the males hind legs, whereas in forced copulation, the male clamps onto the female with one or both hind legs and forcibly inserts his genitalia into hers, while her body is on the ground. In addition, copulation duration was recorded.

### 2.5. Statistical Analyses

Analyses were performed in SYSTAT 13, with the exception of RMA regression, which was accomplished with freely-available online software [22] (http://www.kimvdline.com/professional/rma.html). Transformations were made when data did not meet the assumptions of normality and the homogeneity of variance.

## 3. Results and Discussion

### 3.1. Armament Allometry

RMA regression slopes for each trait and species are shown in Table 1. Spine length and 1° tibial deflection were positively allometric for all species. The two traits absent in *P. marmoratus* but present in the other four species, flange area and 2° tibial deflection, were also positively allometric. Somewhat unexpectedly, femur area was positively allometric only in *P.* ‘Madera’ and *P. marmoratus.* Finally, femur length was isometric in all species.

**Figure 5:**
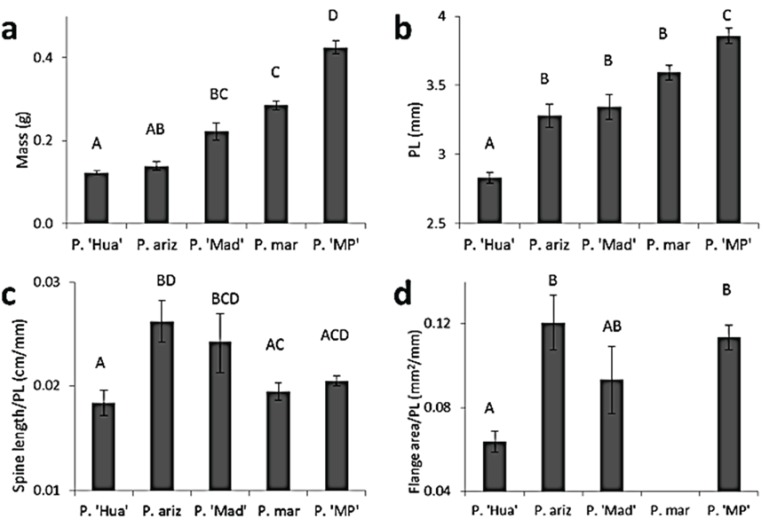

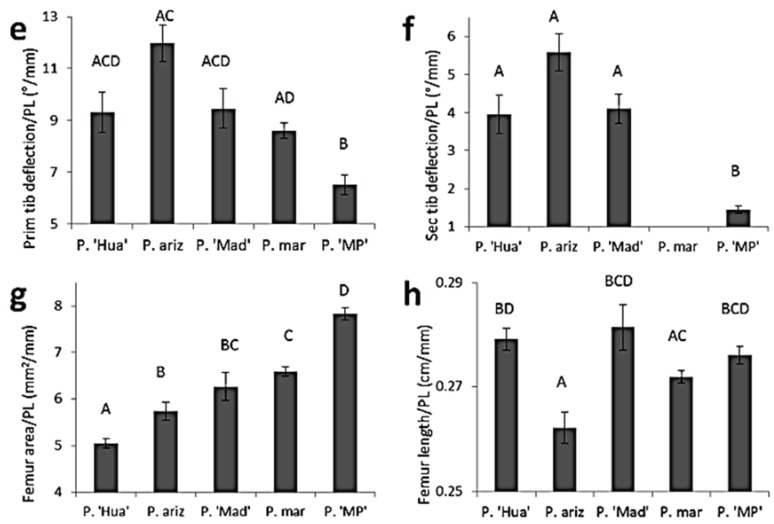
Comparisons of body and leg traits among species of *Pristoceuthophilus*. Letters above bars indicate significantly different species. Species are listed according to increasing mass (**a**) and body size (**b**). Hind leg traits (**c**–**h**) are controlled for body size (pronotum length, PL). Means ± SEM are shown. P. ‘Hua’ = *P*. ‘Huachuca summer’ (*n* = 51); P. ariz = *P. arizonae* (*n* = 12); P. ‘Mad’ = *P.* ‘Madera’ (*n* = 13); P. mar = *P. marmoratus* (*n* = 101); P. ‘MP’ = *P.* ‘Mt. Pinos’ (*n* = 61; prim = primary; sec = secondary; tib = tibial).

The steepness of allometric slopes varied among species (see Table 1, Figure S1). Interestingly, *P.* ‘Huachuca summer,’ the smallest-bodied species (Figure 5), tended to have the steepest allometric slopes for armament traits.

**Table 1:** Reduced major axis (RMA) regressions of hind leg traits on body size for *P.* ‘Huachuca summer’ (*n* = 51), *P. arizonae* (*n* = 12), *P.* ‘Madera’ (*n* = 13), *P. marmoratus* (*n* = 101) and *P. ‘*Mt. Pinos’ (*n* = 61). Prior to RMA analysis, certain variables underwent specific transformations (areas were square root-transformed, and angles were arcsine square root-transformed); all variables subsequently underwent log transformation. If an RMA slope is significantly different from one (shown in bold, *p* ≤ 0.05), a trait is allometric. Species are listed in order of increasing body size (see Figure 5). The values shown are the RMA slope ± SEM (r^2^).

Species	Spine Length	Flange Area	Primary Tibial Deflection	Secondary Tibial Deflection	Femur Area	Femur Length
*P.* ‘Huachuca summer’	**6.24** ± **0.48 (0.71)**	**4.05** ± **0.33 (0.67)**	**6.23** ± **0.56 (0.61)**	**6.44** ± **0.68 (0.45)**	1.13 ± 0.08 (0.74)	0.94 ± 0.07 (0.72)
*P. arizonae*	**3.66** ± **0.68 (0.56)**	**2.77** ± **0.68 (0.40)**	2.09 ± 0.53 (0.36)	**2.48** ± **0.54 (0.54)**	1.07 ± 0.14 (0.82)	0.89 ± 0.12 (0.82)
*P.* ‘Madera’	**5.28** ± **0.70 (0.81)**	**3.85** ± **0.57 (0.76)**	**3.04** ± **0.47 (0.74)**	**2.41** ± **0.60 (0.32)**	1.2 ± 0.1 (0.92)	1.17 ± 0.17 (0.77)
*P. marmoratus*	**4.67** ± **0.29 (0.71)**	–	**2.35** ± **0.13 (0.76)**	–	**1.06** ± **0.03 (0.94)**	1.02 ± 0.04 (0.91)
*P.* ‘Mt. Pinos’	**2.53** ± **0.18 (0.69)**	**2.40** ± **1.15 (0.76)**	**3.62** ± **0.35 (0.43)**	**3.62** ± **0.44 (0.14)**	1.03 ± 0.04 (0.91)	1.02 ± 0.05 (0.84)

### 3.2. Comparisons of Body Size and Relative Armament Size among Species

One-way ANOVAs were used to compare the following traits among all five species: mass, pronotum length and body-size controlled hind leg traits (spine length/pronotum, flange area/pronotum, primary tibial deflection/pronotum, secondary tibial deflection/pronotum, femur area/pronotum and femur length/pronotum). Dividing hind leg traits by pronotum length allowed comparison of the relative investment in these traits among species [23]. All traits were significantly different among species (Figure 5; see Supplementary Table S1 for *F*-values and Table S2 for Tukey’s HSD comparisons). The largest species, *P.* ‘Mt. Pinos,’ had the weakest 1° and 2° tibial deflection; however, no consistent size-related trends prevailed across all species. With respect to the taxonomic issue of whether or not *P.* ‘Huachuca summer’ is a different species than syntopic but phenologically isolated *P. arizonae*, the significant differences in body size (Figure 5b), relative spine length (Figure 5c), relative flange area (Figure 5d), relative femur area (Figure 5g) and relative femur length (Figure 5h) indicate morphological distinctness between the two morphs.

### 3.3. Behavioral Trials

Results of the male-male trials for each species are presented in Table 2. Fights took place in all species. Species differed in their rates of male-male fighting (*G*^2^ = 39.24, df = 3, *p* < 0.001). However, this difference is likely driven by the extremely low rate of fighting in *P.* ‘Mt. Pinos.’ Adult *P*. ‘Mt. Pinos’ individuals barely moved at all during behavioral trials, which could be an artefact of abnormal development in a laboratory setting, as discussed below.

**Table 2:** Male-male trials: frequencies of fighting, copulatory and coercive behaviors performed.

Species	No. of Trials	No. of Trials with:			
		Male-Male Fights	Voluntary Copulation	Attempted Forced Copulation (Unsuccessful)	Successful Forced Copulation
*P.* ‘Huachuca summer’	21	11	15	4	0
*P.* ‘Madera’	16	2	4	0	0
*P. marmoratus*	20	12	2	2	2
*P.* ‘Mt. Pinos’	47	1	2	2	0

Because incentive females were also present during these trials, voluntary copulation and coercion attempts also took place in certain instances. Voluntary copulation was frequent in *P.* ‘Huachuca summer’ male-male trials, although less common in trials of other species. Females were leg-squeezed in all species, except *P.* ‘Madera,’ although no successful forced copulations took place in species other than *P. marmoratus.*

Male-female trials (Table 3) did not resolve whether hind legs successfully serve a second function (mating coercion) in species of *Pristoceuthophilus* other than *P. marmoratus*. Voluntary copulation was most relatively frequent in *P.* ‘Huachuca summer.’ Females were leg-squeezed by males in *P.* ‘Huachuca summer’ and *P.* *marmoratus*, but successful forced copulation only occurred in *P. marmoratus.* No voluntary mating occurred in *P.* ‘Madera,’ and in *P.* ‘Mt. Pinos,’ voluntary copulation took place in only one of 69 trials. (The zeros in the frequency counts make statistical comparisons of the rates of forced copulation between species impossible.)

**Table 3:** Male-female trials: frequencies of voluntary and coercive copulatory behaviors performed.

Species	Total No. of trials	Total No. of Trials with				
		Copulation Attempted (Successful/Unsuccessful)	Unsuccessful Attempted Copulation		Successful Copulation	
			*Forceful*	*Voluntary*	*Forceful*	*Voluntary*
*P.* ‘Huachuca summer’	16	14	3	0	0	11
*P.* ‘Madera’	11	0	0	0	0	0
*P. marmoratus*	101	33	15	0	4	14
*P.* ‘Mt. Pinos’	69	1	0	0	0	1

**Figure 6:**
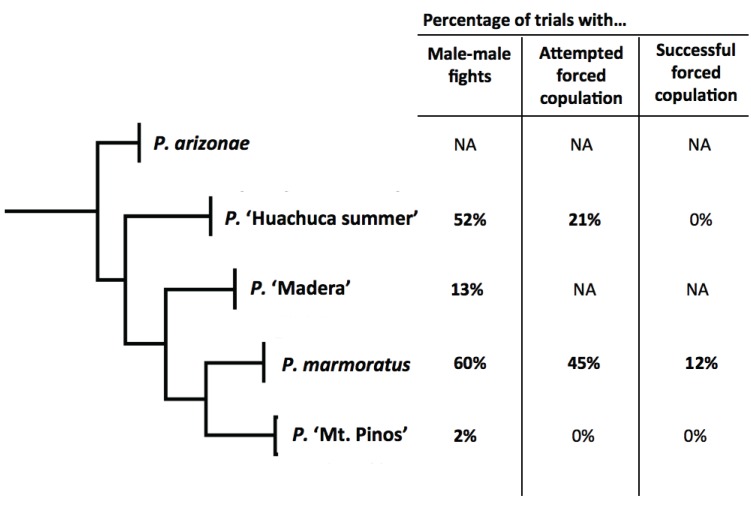
Rates of fighting and coercive behaviors in four species of *Pristoceuthophilus*. For male-male fights, percentages were determined by dividing the number of trials with observed fights by the total number of trials. For attempted and successful forced copulation, percentages were determined by dividing the number of trials with attempted or successful forced copulation by the total number of trials with copulation attempted (voluntary + forceful). No behavioral trials were performed for *P. arizonae.* In *P.* ‘Madera,’ no male-female trials resulted in any copulation attempt; thus, the results are inconclusive.

Combined, the behavioral trials provide evidence of a male-male agonistic function in all species for which trials were performed and the suggestion of a sexual coercion function in *P.* ‘Huachuca summer’ and perhaps in *P.* ‘Mt. Pinos’ (see male-male behavioral trials) (Figure 6). Thus, armaments likely serve dual purposes in *P.* ‘Huachuca summer’ and in *P.* ‘Mt. Pinos,’ as well as in *P. marmoratus.* Note: these are the first data to be reported on fighting and mating behavior in these species (other than *P. marmoratus*) and are limited in uneven sample sizes (*P.* ‘Madera’ has an especially small sample size) and potentially confounded in *P.* ‘Mt. Pinos’ (see Section 3.7 below). They should be interpreted cautiously.

### 3.4. General Observations of Voluntary Copulatory Behavior

Voluntary copulatory behavior in all species was similar to that previously recorded for *P. marmoratus* [10]. Males invited females to mount them by spreading their hind legs and pushing their abdomen back towards the female. Females often antennated the posterior portion of the male abdomen at this point, possibly related to the detection of olfactory cues [24].

Copulation duration, recorded as the time from the beginning to the end of genital coupling, was highly variable among species (non-parametric Kruskal–Wallis *K*_3,45_ =34.13, *p* < 0.001). Mean ± SE voluntary copulation durations were as follows (species listed from smallest-bodied to largest-bodied): *P.* ‘Huachuca summer,’ 17.1 ± 0.8 minutes (*n* = 26); *P.* ‘Madera,’ 145.7 ± 18.0 min (*n* = 4); *P. marmoratus*, 62.8 ± 5.5 min (*n* = 14); *P.* ‘Mt. Pinos,’ 14.1 ± 0.5 min (*n* = 3). Copulation durations were significantly different between all species (see Supplementary Table S3 for the *p*-values from pairwise comparisons).

### 3.5. Status of *P*. ‘Huachuca Summer’

According to the leading *Pristoceuthophilus* expert, the late Ted Cohn, only two species of *Pristoceuthophilus* occur in Arizona, *P. arizonae* and *P*. ‘Madera’ (“*P. leptodrilus*” in T.J. Cohn’s notes). In this study, we provide evidence that Arizona-dwelling *P.* ‘Huachuca summer’ is morphologically and genetically distinct, not only from syntopic *P. arizonae* and sympatric *P.* ‘Madera,’ but also from all other species of *Pristoceuthophilus* in this study. *P.* ‘Huachuca summer’ had a recognizably different pseudosternite and unusually high allometric slopes for armament traits. *P.* ‘Huachuca summer’ also differed from *P. arizonae* and *P.* ‘Madera’ in relative investment in several armament traits.

### 3.6. Comparisons of Allometries and Armaments

Traits that were previously shown to be positively allometric in *P. marmoratus* were also positively allometric in the four other species in this study (with the exception of femur area, which was inconsistent). The two new traits introduced in this study, flange area and 2° tibial deflection, also proved to be positively allometric. Biomechanical analyses would be necessary to show whether these two additional traits improve leg-squeezing efficacy. Based on the 16s phylogeny (see Figure 3), the most parsimonious explanation for the absence of these two traits in *P. marmoratus* would be a loss of these traits from the ancestral state. Interestingly, *P. marmoratus*, engages in frequent male-male fights (about 60% of male-male encounters ended in a fight; Table 2) and is the only species shown to successfully force-copulate. Somewhat unsettlingly, it would appear that the least armed species (in terms of total number of armaments) experiences the greatest selection for agonistic and sexually antagonistic leg use. More behavioral trials in the other species of *Pristoceuthophilus* are necessary to resolve this unintuitive conclusion.

Species showed variation in the allometric slopes of armament traits. Allometric slopes tended to be highest for *P.* ‘Huachuca summer,’ the smallest species. Some authors have argued that steeper allometric slopes of armaments indicate greater marginal fitness payoffs when larger individuals disproportionately invest in armaments [15,16]. In addition, allometric slopes may be increased by the strength of sexual selection on a trait [16,17]; however, this pattern may not be universal (see [25]). The high rate of male-male fighting in *P.* ‘Huachuca summer’ (about 50% of male-male encounters ended in a fight; Table 2) seems to support a strong sexual selection pressure, although the same fails to hold true for *P. marmoratus*, which exhibited much weaker allometric slopes despite engaging in male-male fights at a higher rate than *P.* ‘Huachuca summer.’ Allometric slope steepness was not consistent among armaments for other species.

Relative armament investment also showed little in the way of trends among species. The only somewhat consistent pattern was that *P.* ‘Mt. Pinos’ tended to have the smallest relative armament investment (for spine length and 1° and 2° tibial deflection), but this could be an artefact of suboptimal dietary rearing conditions (P. ‘Mt. Pinos’ was the only species reared in the lab; all other species were wild-caught as adults). The condition-dependence of these traits in *P. marmoratus* [14] would likely also occur in *P.* ‘Mt. Pinos,’ although not being directly tested here. Thus, an insufficient diet would likely produce small weaponry traits in *P.* ‘Mt. Pinos.’

### 3.7. Dual-Purpose Armaments in Other Pristoceuthophilus?

Before Haley and Gray’s (2012) study [10], no one knew for what the elaborately-modified hind legs of male *Pristoceuthophilus* were used. Haley and Gray’s work demonstrated not only that the legs function in male-male fighting, as might be expected, but also that the legs facilitate mating coercion. Although broadly functional weapons exist in other taxa, such as the aculeus of scorpions, which is used to inject venom into prey, foes and mates alike [26], there are few cases of weapons being used in functionally identical manners in both intra- and inter-sexual interactions. The apparent rareness of such a “dual-purpose armament” among all animals prompted us to explore how widespread this trait might be among morphologically similar congeners.

This study revealed that male hind legs serve the first purpose, male-male fighting, in three additional species of *Pristoceuthophilus*. Leg-mediated sexual coercion attempts were present in two species, *P.* ‘Huachuca summer’ and *P.* ‘Mt. Pinos,’ but never ended in successful forced copulation. Because of this, we cannot conclusively say that hind leg armaments are dual-purpose in these species. However, because of low rates of forced copulation in *P. marmoratus* (4 of 101 trials), small sample sizes in *P.* ‘Huachuca summer’ and in *P.* ‘Madera’ probably precluded successful observation of this behavior. Sample sizes in *P.* ‘Mt. Pinos’ were very large: 47 male-male trials and 69 male-female trials. Yet, out of all of these trials, only three voluntary copulations, two male leg-squeezes of females and one male-male fight occurred. However, we believe that the general lack of mating and fighting behavior in *P.* ‘Mt. Pinos’ is most likely explained by the fact that, unlike the other species in this study, *P.* ‘Mt. Pinos’ individuals were collected from the field as juveniles rather than as adults. Lab rearing may have had detrimental effects on this species: approximately half of females exhibited broken ovipositors as adults. Males appeared externally normal, but perhaps were hormonally abnormal or completely deterred by female deformities. *Pristoceuthophilus* species are sensitive to laboratory conditions. Unlike *Gryllus* field crickets, which oviposit in the lab without hesitation, *Pristoceuthophilus* crickets have never laid eggs in the lab, despite being offered a number of egg-laying substrates (sand, potting soil, dirt from the place they were collected). It appears that nymphal development might also not occur normally in the laboratory conditions used.

Despite some problems with sample sizes and nymphal developmental, we still observed attempts at forced copulation in species of *Pristoceuthophilus* other than *P. marmoratus*. The fact that the rate of leg-mediated forced copulation attempts was much lower than the rate of leg-mediated male fights (Figure 6) suggests that hind leg armaments’ primary function is male-male competition and that sexual coercion is a secondary exaptation [27]; a true test of this question will require much greater taxon sampling and phylogenetic reconstructions.

## 4. Conclusions

Use of modified hind legs in “nutcracker” species of *Pristoceuthophilus* in male-male fighting appears common in all species so far examined. The “dual-purpose” use of those armaments in a mating context also appears commonplace across species, but less frequent within species. We interpret this as support for the idea that the use of the modified legs in a mating context is a secondary function that appeared evolutionarily after the use of legs in male-male fighting. The strong positive allometries of male armaments indicates strong selection, as could occur from sexual selection via either male-male or male-female interactions. However, because our other work with *P. marmoratus* shows that the males most likely to use legs to capture and hold females are the smaller males with less armaments in poorer condition [11], we consider it more likely that it was selection in the context of male-male fighting that led to the elaboration of the “nutcracker” leg armature.

## Supplementary Files

Supplementary File 1
