# Supplementary material for: Male Armaments and Reproductive Behavior in “Nutcracker” Camel Crickets (Rhaphidophoridae, Pristoceuthophilus)
Source: Insects. 2015 Jan 7;6(1):85–99. doi: 10.3390/insects6010085 (PMC4553529; doi:10.3390/insects6010085)
Supplement: Supplementary File 1 [file insects-06-00085-s001.pdf]

Supplementary Materials

Table S1. Single-factor ANOVAs comparing traits of the five species. Hind leg traits are body size-controlled. See Table S2 for between-species pair comparisons (Tukey's HSD).

**Trait**	***F***	**df**	***p***
Mass	71.89	4, 236	**<0.001**
Pronotum (body size)	50.17	4, 236	**<0.001**
Spine length/pronotum	4.36	4, 236	**0.002**
Flange area/pronotum	13.97	3, 137	**<0.001**
1° angle of tibial deflection/pronotum	8.34	4, 236	**<0.001**
2° angle of tibial deflection/pronotum	17.49	3, 137	**<0.001**
Femur area/pronotum	66.01	4, 236	**<0.001**
Femur length/pronotum	6.45	4, 236	**<0.001**

Table S2. Tukey's HSD comparisons of body and leg traits between species (Pariz = *P. arizonae*; PHua = *P.* 'Huachuca summer'; PMad = *P.* 'Madera'; Pmar = *P. marmoratus*; PMP = *P.* 'Mt. Pinos').

**Trait**	**Species 1**	**Species 2**	**Difference**	***p***
Mass	Pariz	PMP	−0.286	**<0.001**
	Pariz	PHua	0.017	0.985
	Pariz	PMad	−0.082	0.205
	Pariz	Pmar	−0.145	**<0.001**
	PMP	PHua	0.303	**<0.001**
	PMP	PMad	0.204	**<0.001**
	PMP	Pmar	0.141	**<0.001**
	PHua	PMad	−0.099	**0.009**
	PHua	Pmar	−0.162	**<0.001**
	PMad	Pmar	−0.063	0.172
Pronotum	Pariz	PMP	−0.578	**<0.001**
	Pariz	PHua	0.448	**0.004**
	Pariz	PMad	−0.064	0.995
	Pariz	Pmar	−0.313	0.083
	PMP	PHua	1.026	**<0.001**
	PMP	PMad	0.515	**<0.001**
	PMP	Pmar	0.265	**0.001**
	PHua	PMad	−0.512	**<0.001**
	PHua	Pmar	−0.761	**<0.001**
	PMad	Pmar	−0.249	0.224
Spine length/pronotum	Pariz	PMP	0.006	0.072
	Pariz	PHua	0.008	**0.005**
	Pariz	PMad	0.002	0.957
	Pariz	Pmar	0.007	**0.017**
	PMP	PHua	0.002	0.516
	PMP	PMad	−0.004	0.396
	PMP	Pmar	0.001	0.926
	PHua	PMad	−0.006	0.055
	PHua	Pmar	−0.001	0.905
	PMad	Pmar	0.005	0.158

Table S2. *Cont.*

Trait	Species 1	Species 2	Difference	*p*
Flange area/pronotum	Pariz	PMP	0	0.953
	Pariz	PHua	0.001	**<0.001**
	Pariz	PMad	0	0.394
	PMP	PHua	0.001	**<0.001**
	PMP	PMad	0	0.426
	PHua	PMad	0	0.121
1° angle of tibial deflection/pronotum	Pariz	PMP	5.466	**<0.001**
	Pariz	PHua	2.678	0.137
	Pariz	PMad	2.521	0.401
	Pariz	Pmar	3.373	**0.021**
	PMP	PHua	−2.788	**<0.001**
	PMP	PMad	−2.945	0.056
	PMP	Pmar	−2.093	**0.006**
	PHua	PMad	−0.157	1
	PHua	Pmar	0.695	0.821
	PMad	Pmar	0.852	0.933
2° angle of tibial deflection/pronotum	Pariz	PMP	4.133	**<0.001**
	Pariz	PHua	1.629	0.133
	Pariz	PMad	1.482	0.391
	PMP	PHua	−2.504	**<0.001**
	PMP	PMad	−2.65	**0.001**
	PHua	PMad	−0.147	0.997
Femur area/pronotum	Pariz	PMP	−0.021	**<0.001**
	Pariz	PHua	0.007	**0.004**
	Pariz	PMad	−0.005	0.622
	Pariz	Pmar	−0.009	**0.024**
	PMP	PHua	0.028	**<0.001**
	PMP	PMad	0.016	**<0.001**
	PMP	Pmar	0.012	**<0.001**
	PHua	PMad	−0.012	**<0.001**
	PHua	Pmar	−0.015	**<0.001**
	PMad	Pmar	−0.003	0.748
Femur length/pronotum	Pariz	PMP	−0.014	**0.005**
	Pariz	PHua	−0.017	**<0.001**
	Pariz	PMad	−0.019	**0.002**
	Pariz	Pmar	−0.01	0.097
	PMP	PHua	−0.003	0.696
	PMP	PMad	−0.005	0.645
	PMP	Pmar	0.004	0.327
	PHua	PMad	−0.002	0.981
	PHua	Pmar	0.007	**0.014**
	PMad	Pmar	0.009	0.096

Table S3. Pairwise comparisons of voluntary copulation duration between species using the Dwass–Steel–Critchlow–Fligner Test (PHua = *P.* ‘Huachuca summer’; PMad = *P.* ‘Madera’; Pmar = *P. marmoratus*; PMP = *P.* ‘Mt. Pinos’).

Species 1	Species 2	Test Statistic	*p*
PMP	PHua	40.92	<0.001
PMP	PMad	5.00	**0.002**
PMP	Pmar	19.88	**<0.001**
PHua	PMad	−28.63	**<0.001**
PHua	Pmar	−6.02	**<0.001**
PMad	Pmar	8.81	**<0.001**

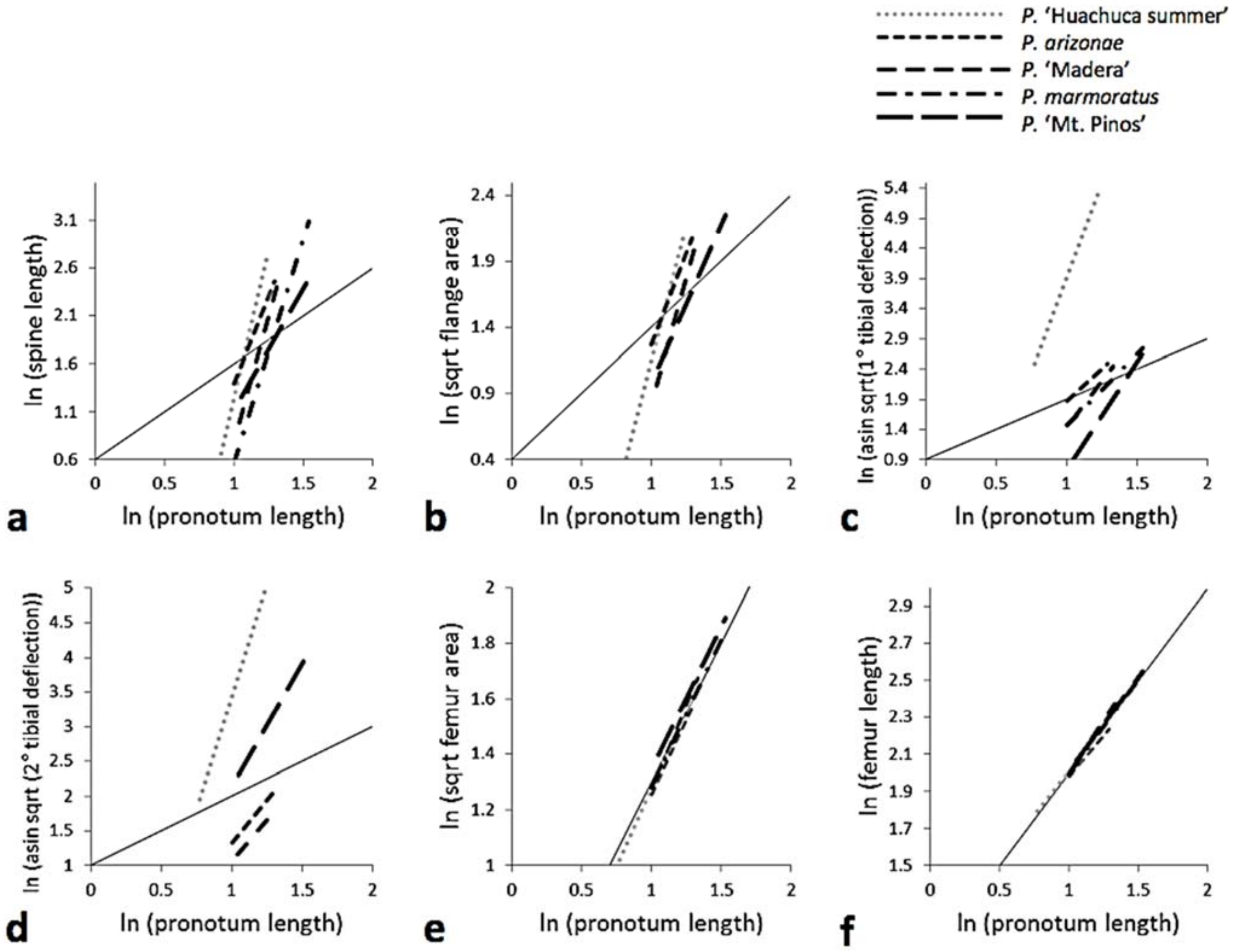

Figure S1. RMA regression slopes showing log-transformed pronotum length *versus* log-transformed (**a**) spine length, (**b**) square root-transformed flange area, (**c**), arcsine square root-transformed primary tibial deflection, (**d**) arcsine square root-transformed secondary tibial deflection, (**e**) square root-transformed femur area and (**f**) femur length. The solid line is the line of isometry (slope = 1), while each dashed line represents a different species (see the legend). Lines are drawn such that the endpoints encompass the observed range of variation in pronotum length. *Pristoceuthophilus marmoratus* lacks a flange (**b**) and a secondary tibial deflection (**d**) and, thus, does not appear in those graphs.
